# The digital use of simulated patients in times of the corona pandemic – considerations and proposals

**DOI:** 10.3205/zma001386

**Published:** 2020-12-03

**Authors:** Tim Peters, Christian Thrien, German Association for Medical Education, committee for simulated patients

**Affiliations:** 1 University of Applied Sciences, Department of Nursing Sciences, Bochum, Germany; 2 University of Cologne, Cologne Interprofessional Skills Lab and Simulation Centre, Cologne, Germany

**Keywords:** simulated patient, human simulation, SARS-CoV-2, Covid 19, safety, learning objectives, constructive alignment

## Abstract

The corona pandemic has posed major challenges for teaching with simulated persons (SPs), which usually requires the physical presence of the participants. Within a short period of time, a large number of individual solutions were developed. The committee “Simulated Persons” of the Society for Medical Education has developed considerations and proposals in five areas to meet the qualitative challenges of the method.

First and foremost, the safety of the SPs is at stake, both in terms of infection prevention and role-related stress to which the SPs are now exposed at home alone instead of the usual setting, where they are in a teaching building with the connection to the staff on site. Furthermore, it should be noted that the changed framework conditions also require a reflection on behalf of the learning objectives, since not all teaching scenarios with SPs can be transferred from a real setting to a digital environment. Furthermore, even under corona conditions, the constructive alignment must not be disregarded, i.e. the question of testability must be considered from the very beginning. Aspects of the technical infrastructure for all participants and compliance with data protection requirements must also be considered. Last but not least, the forced changes are also an opportunity to take a proactive approach to the topic of telemedicine in teaching.

## Zusammenfassung

Die Corona-Pandemie hat die Lehre mit Simulationspersonen (SPs), die üblicherweise die physische Präsenz der Beteiligten voraussetzt, vor große Herausforderungen gestellt. In kurzer Zeit wurde eine Vielzahl individueller Lösungen entwickelt. Der Ausschuss „Simulationspersonen“ der Gesellschaft für Medizinische Ausbildung hat in fünf Bereichen Überlegungen und Vorschläge erarbeitet, um den qualitativen Herausforderungen an die Methode zu begegnen.

An erster Stelle geht es um die Sicherheit der SPs, sowohl in Bezug auf den Infektionsschutz als auch auf rollenbedingte Belastungen, denen die SPs nun allein zuhause statt im gewohnten Setting eines Lehrgebäudes mit der Anbindung an das Personal vor Ort ausgesetzt sind. Im Weiteren ist darauf zu achten, dass die geänderten Rahmenbedingungen auch eine Reflexion der Lernziele erfordert, da nicht alle Lehrveranstaltungen mit SPs vom realen Setting in eine digitale Umgebung übertragen werden können. Außerdem darf auch unter Corona-Bedingungen das constructive alignment nicht außer Acht gelassen werden, also sollte die Frage der Prüfbarkeit von Anfang an mitgedacht werden. Auch Aspekte der technischen Infrastruktur bei allen Beteiligten und die Einhaltung der Datenschutzerfordernisse müssen beachtet werden. Zu guter Letzt sind die erzwungenen Veränderungen auch eine Chance, das Thema Telemedizin in der Lehre offensiv anzugehen.

## Introduction

Simulated patients (SPs) are an essential part of medical education and training [1], [2], [3], [4]. The corona pandemic has pushed digital teaching to a degree that was hardly imaginable before. This also applied to the use of SPs, although the digital character is initially conceptually opposed to the typical aspects of simulating a contact in physical presence. Nevertheless, many individual solutions for this supposed contradiction were found in a short time. In view of this special situation, the Simulated Persons Committee of the Society for Medical Education has developed some considerations and proposals to meet the qualitative challenges of the method.

## Safety

Direct SP contacts should be avoided where possible in times of a pandemic. Likewise, SP-trainings or SP-courses should be cancelled or conducted digitally. In this context, reference is also made to point 1 of the Committee's position paper [5], which calls for a safe working environment. Nevertheless, we see that it can be useful in justified cases and is sometimes required by responsible authorities to use SPs. This could include both assignments in the training buildings and assignments in digitally mediated form. In order not to give up the claim to a safe working environment even then, at least the following two aspects must be considered:

1. Compliance with infection protection: e.g. wearing protective clothing, mouth and nose protection, etc. depending on the implementation of the specified rules.
2. Protection from stress due to the nature of the role: Professional actors are usually more experienced than laymen due to their professional training, but certain simulations such as delivering bad news are naturally associated with a higher risk of stress. During teaching with SPs in presence, special attention is usually paid to ensure that the SPs succeed in stepping out of the role. This could be more difficult when such a role is presented at the private home. In addition, SPs are alone after a virtual simulation. Here, special attention should be paid to who is used in such simulations and how easy SP-trainers can be approached after the simulation.

## Orientation on learning objectives

When transferring SP encounters to the digital world, it should be noted that the learning objectives and competences to be achieved are decisive. Learning objectives that relate to the structuring of conversations or the phrasing of specific questions can surely be taught and learned with SPs digitally. Aspects such as non-verbal behavior or dealing with grief are strongly changed by digitization. This does not mean that nothing can be learned in this way. What is learned, however, applies precisely in this (digital) context and cannot simply be transferred to face-to-face interactions. All in all, the aim should not be to transpose SP-based teaching digitally to any applicable extent, but rather to do so with care and a sense of proportion with regard to the learning objectives to be achieved.

## Constructive alignment

In the case of digital SP formats, it should be considered whether these situations occur in a similar form later in examinations or whether they are relevant in practice [6]. From the training of delivering bad news with an SP without the possibility of physical attention and in a purely digital form, we would expect only moderately good preparation for a later OSCE station or for delivering a message later on in practice. For difficult conversation situations under digital conditions, however, this teaching format could possibly already be an adequate preparation. For structuring conversations or recognizing emotional aspects, on the other hand, it would be more appropriate to practice this digitally so that they can be retrieved later in exams or practice in a manner appropriate to the situation. If digital SP encounters are planned, SPs must be trained for this, since the processes and case scenarios will change and the aspects of data protection and technology listed below must be considered.

## Infrastructure & privacy

For digital SP encounters, both sides need the technical possibilities of an exchange, including a corresponding – if possible not privately used – account. In addition, it must be noted whether the software used complies with German or European data protection regulations. However, there are also further questions: Can both SPs and students perform their simulations while they are sitting at home in their living room or office - with the consequence that the counterpart is also watching? How to deal with the fact that uninvolved third parties on the side of the SP or the students are aware of or even comment on the conversation due to the home office situation? Alternatively the SPs would sit in a central unit, which also includes a variety of contacts and thus also risks. Overall, the SPs should be informed about aspects such as technology, data protection and confidentiality, as should the students, and if necessary, consent should be obtained.

## Telemedicine as an opportunity!

Finally, Corona forces us to deal with a topic that will be on the agenda more often in the future. Telemedicine, which is often invoked but rarely addressed in medical teaching, can now become a new focus of learning due to necessity. Without reacting to developments with a time delay, this time we can use digital SP encounters to prepare students for future digital contacts with patients which will happen anyway and change the healthcare system. Here we now have the (forced) possibility and opportunity to develop teaching concepts and training scenarios that we can use beyond the corona crisis.

## Competing interests

The authors declare that they have no competing interests. 
